# A cohort study: temporal trends in prevalence of antecedents, comorbidities and mortality in Aboriginal and non-Aboriginal Australians with first heart failure hospitalization, 2000–2009

**DOI:** 10.1186/s12939-015-0197-4

**Published:** 2015-08-12

**Authors:** Tiew-Hwa Katherine Teng, Judith M. Katzenellenbogen, Joseph Hung, Matthew Knuiman, Frank M. Sanfilippo, Elizabeth Geelhoed, Dawn Bessarab, Michael Hobbs, Sandra C. Thompson

**Affiliations:** Western Australian Centre for Rural Health, University of Western Australia (UWA), Perth, Australia; School of Medicine & Pharmacology, Sir Charles Gairdner Hospital Unit, UWA, Perth, Australia; School of Population Health, UWA, Perth, Australia; Centre for Aboriginal Medical and Dental Health, UWA, Perth, Australia

**Keywords:** Heart failure, Risk factors, Mortality, Indigenous, Aboriginal

## Abstract

**Background/objectives:**

Little is known about trends in risk factors and mortality for Aboriginal Australians with heart failure (HF). This population-based study evaluated trends in prevalence of risk factors, 30-day and 1-year all-cause mortality following first HF hospitalization among Aboriginal and non-Aboriginal Western Australians in the decade 2000–2009.

**Methods:**

Linked-health data were used to identify patients (20–84 years), with a first-ever HF hospitalization. Trends in demographics, comorbidities, interventions and risk factors were evaluated. Logistic and Cox regression models were fitted to test and compare trends over time in 30-day and 1-year mortality.

**Results:**

Of 17,379 HF patients, 1,013 (5.8 %) were Aboriginal. Compared with 2000–2002, the prevalence (as history) of myocardial infarction and hypertension increased more markedly in 2006–2009 in Aboriginal (versus non-Aboriginal) patients, while diabetes and chronic kidney disease remained disproportionately higher in Aboriginal patients. Risk factor trends, including the Charlson comorbidity index, increased over time in younger Aboriginal patients. Risk-adjusted 30-day mortality did not change over the decade in either group. Risk-adjusted 1-year mortality (in 30-day survivors) was non-significantly higher in Aboriginal patients in 2006–2008 compared with 2000–2002 (hazard ratio (HR) 1.44; 95 % CI 0.85-2.41; p-trend = 0.47) whereas it decreased in non-Aboriginal patients (HR 0.87; 95 % CI 0.78-0.97; p-trend = 0.01).

**Conclusions:**

Between 2000 and 2009, the prevalence of HF antecedents increased and remained disproportionately higher in Aboriginal (versus non-Aboriginal) HF patients. Risk-adjusted 1-year mortality did not improve in Aboriginal patients over the period in contrast with non-Aboriginal patients. These findings highlight the need for better prevention and post-HF care in Aboriginal Australians.

## Introduction

Indigenous minorities in affluent countries like Australia, Canada, New Zealand and the United States have considerably lower life expectancies compared with the non-Indigenous populations [1]. Excess burden of cardiovascular disease (CVD), including heart failure (HF), is a principal contributor to these disparities [2–5]. This is particularly pertinent in Australia, where Indigenous (Aboriginal and Torres Strait Islander, hereafter Aboriginal) people, colonised predominantly since the early 1800s, have a life expectancy gap of 10 years [6] and a median age of 22 years compared with 37 years in other Australians. Despite representing only 3 % (670,000 people) of the Australian population, Aboriginal experience disadvantage across virtually all health and of socio-economic indicators, including education, employment and income [6].

In Australia, CVD accounts for about a quarter of the differential disease burden in the Aboriginal population [7]. Prevalence of CVD is reported to be 1.3–2.5-fold higher among Aboriginal people compared to non-Aboriginal people [2, 3]. Many modifiable lifestyle risk factors (smoking, physical inactivity, poor nutrition) and CVD risk factors (diabetes, chronic kidney disease, overweight/obesity, hypertension) are more common in Aboriginal than non-Aboriginal populations [2, 3], predisposing them to the development of CVD [8].

Heart failure (HF) is a common, morbid and costly disease with poor prognosis [9]. It is a chronic, progressive clinical syndrome that is characterised by underlying structural or functional impairment of ventricular filling or ejection of blood [10]. The cardinal manifestations are fatigue and dyspnoea at rest or during physical activity, and often fluid retention, pulmonary congestion and/or peripheral oedema [10].

HF is a sequelae of many CVD, including ischaemic heart disease (IHD), hypertension, atrial fibrillation, valvular heart disease and idiopathic cardiomyopathy, often causing death [9]. Diabetes, obesity, chronic kidney disease and rheumatic heart disease, which also disproportionately affect Aboriginal populations [11, 12], are other risk factors for HF [9].

Earlier longitudinal population-based studies from Australia [13, 14] and other high socio-economic countries [15–17] have reported sustained declines in short-term and long-term mortality after initial hospitalization for HF, corresponding to the era of better cardiovascular prevention and evidence-based treatment for HF since the mid-1990s. However, there is little literature pertaining to Aboriginal populations. In a recent study, we reported that for the period of 2000–2009, the incidence rate overall of first hospitalization for HF was three to five times higher for Aboriginal men and women than non-Aboriginal [18].

The prevalence and magnitude of risk factors for HF drive its incidence. Thus, understanding trends in risk factor prevalence is important, with implications for prevention and future service demand. Furthermore, differences in mortality trends between Aboriginal and non-Aboriginal patients are measures of quality of cardiovascular care and secondary prevention of HF. However, there is little literature pertaining to trends in risk factors and mortality associated with HF in the Australian Aboriginal population. The purpose of this study was to examine and compare the trends in antecedent risk factors, comorbidities and mortality in Aboriginal (versus non-Aboriginal) patients with first hospitalization for HF between years 2000 and 2009.

## Methodology

### Setting

Western Australia (WA), the largest state of Australia, has a land area twelve times that of United Kingdom, and is the home of the third highest number of Aboriginal Australians (13.2 %), of which an estimated 41 % live in remote locations.

### Data sources and study cohort

Since the 1970s, the WA Hospital Morbidity Data Collections (HMDC) have recorded principal and secondary (up to 20) discharge diagnoses for all patients discharged from all (149) public and private hospitals in WA [19]. The HMDC is a core dataset of the WA Data Linkage System and is routinely linked to other administrative datasets, e.g. the state death registry [19].

Using methods described previously [14, 18], we identified a population-based cohort using the HMDC comprising all WA residents, aged 20–84 years, who had their first (index) HF inpatient hospitalization between January 2000 and December 2009. Patients were excluded if they had any admission for HF in the previous 10 years from their index admission. The International Classification of Diseases, Ninth Revision (ICD9) and Tenth Revision (ICD10) diagnostic codes (including 428× or I50×), were used to code HF, as a principal discharge diagnosis, or a secondary discharge diagnosis of HF [only where the principal discharge diagnosis was a cardiovascular condition, excluding acute myocardial infarction (AMI)].

The coding for HF as a principal diagnosis (*n* = 1,006) in the WA HMDC has been validated against the Boston diagnostic criteria [comprising categories of history (dyspnoea), physical examination and chest radiography), with a positive predictive value of more than 92 % for ‘definite’ HF [20]. Furthermore, discharge evidence-based medications for HF and echocardiography reports in the cohort were also examined in the validation cohort [21].

### Aboriginal status

Aboriginal status is routinely collected through self-identification for patients admitted for hospital treatment, although this is known to be under-recorded [22]. Since 2002, the coding of Aboriginal status in the HMDC exceeds 90 % [23]. Since 2010, the National best practice guidelines for collecting Indigenous status in health datasets have been implemented to ensure consistency and accurate recording of Indigenous status to improve the reliability of the data [22]. Furthermore, to reduce the impact of under-recording of Aboriginal status, a patient was defined in our study as being Aboriginal if at least 25 % of all hospital admissions (since 1980) for that patient had been coded as Aboriginal or Torres Strait Islander.

### Antecedents of HF, comorbidities and procedures

Antecedents of HF and comorbidities (Table 1) were identified using a fixed 5-year look-back or were concurrent with the index HF hospitalization. The Charlson comorbidity index [24], a composite measure of comorbidity burden, was calculated using the modified Deyo algorithm [25]. Coronary angiography, percutaneous coronary intervention (PCI) or coronary artery bypass grafting (CABG) were similarly identified.

**Table 1:** Characteristics of Aboriginal and non-Aboriginal patients with a ‘first-ever’ admission for heart failure between 2000–2009

	Aboriginal (*n* = 1,013)				Non-Aboriginal (*n* = 16,366)			
Description	2000–2002 *n* = 276	2003–2005 *n* = 305	2006–2009 *n* = 432	*p*-value	2000–2002 *n* = 4,855	2003–2005 *n* = 4,872	2006–2009 *n* = 6,639	*p*-value
Women, n (%)	136 (49.3)	153 (50.2)	224 (51.9)	0.78	2,102 (43.3)	2,003 (41.1)	2,726 (41.1)	0.03
Mean age ± SD (years)	53.8 ± 14.3	53.0 ± 13.9	54.9 ± 14.1	0.15	71.2 ± 10.8	71.0 ± 11.5	70.8 ± 11.8	0.03
Age groups, n (%)								
• 20–34 years	36 (13.0)	33 (10.8)	35 (8.1)		46 (0.9)	54 (1.1)	80 (1.2)	
• 35–49 years	68 (24.6)	85 (27.9)	119 (27.6)	0.36	190 (3.9)	240 (4.9)	335 (5.1)	0.01
• 50–64 years	99 (35.9)	117 (38.4)	159 (36.8)		839 (17.3)	866 (17.8)	1,253 (18.9)	
• 65–84 years	73 (26.5)	70 (23.0)	119 (27.6)		3,780 (77.9)	3,712 (76.2)	4,971 (74.9)	
Urban/Rural, n (%)								
• Rural	217 (78.6)	230 (75.4)	325 (75.2)	0.54	1,111 (22.9)	1,099 (22.6)	1,521 (22.9)	0.89
• Urban	59 (21.4)	75 (24.6)	107 (24.8)		3,743 (77.1)	3,773 (77.4)	5,117 (77.1)	
SEIFA –quintiles, n (%)								
• 1^st^ quintile (most disadvantaged)	148 (53.6)	150 (49.3)	194 (45.0)	0.04	228 (4.7)	193 (4.0)	269 (4.1)	
• 2^nd^ quintile	25 (9.1)	28 (9.2)	41 (9.5)		815 (16.8)	793 (16.3)	1,076 (16.2)	
• 3^rd^ quintile	72 (26.1)	79 (26.0)	124 (28.8)		1,768 (36.5)	1,762 (36.2)	2,498 (37.7)	0.001
• 4^th^ quintile	27 (9.8)	32 (10.5)	64 (14.9)		929 (19.2)	1,049 (21.6)	1,448 (21.9)	
• 5^th^ quintile	<5 (1.5)	15 (4.9)	8 (1.9)		1,100 (22.7)	1,068 (22.0)	1,333 (20.1)	
With private medical insurance, n (%)	<5 (1.1)	6 (2.0)	5 (1.2)	0.58	1,383 (28.5)	1,485 (30.5)	2,230 (33.6)	<0.001
Length of stay, days ± SD	5.1 ± 5.9	5.3 ± 6.2	4.6 ± 4.9	0.12	7.3 ± 15.5	7.3 ± 9.5	6.6 ± 8.3	0.001
Comorbidities, n (%)^a^								
All ischaemic heart disease^b^	117 (42.4)	134 (43.9)	182 (42.1)	0.88	2,516 (51.8)	2,368 (48.6)	3,004 (45.3)	<0.001
Acute myocardial infarction	37 (13.4)	58 (19.0)	91 (21.1)	0.04	781 (16.3)	760 (15.6)	1,143 (17.2)	0.06
Unstable angina	35 (12.7)	37 (12.1)	60 (13.9)	0.77	846 (17.4)	761 (15.6)	733 (11.0)	<0.001
Hypertension	164 (59.4)	192 (62.9)	312 (72.2)	0.001	2,575 (53.0)	2,568 (52.7)	3,791 (57.1)	<0.001
Atrial fibrillation	59 (21.4)	50 (16.4)	102 (23.6)	0.06	2,001 (41.2)	2,108 (43.3)	2,951 (44.5)	0.01
Diabetes	164 (59.4)	191 (62.6)	268 (62.0)	0.70	1,407 (29.0)	1,459 (30.0)	2,158 (32.5)	<0.001
Chronic kidney disease	96 (34.8)	114 (37.4)	179 (41.4)	0.19	944 (19.4)	941 (19.3)	1,495 (22.5)	<0.001
Renal failure	38 (13.8)	42 (13.8)	53 (12.3)	0.78	143 (3.0)	129 (2.7)	191 (2.9)	0.65
COPD	83 (30.1)	89 (29.2)	119 (27.6)	0.75	1,200 (24.7)	1,021 (21.0)	1,234 (18.6)	<0.001
Cerebrovascular disease	25 (9.1)	19 (6.2)	34 (7.9)	0.44	618 (12.7)	483 (9.9)	616 (9.3)	<0.001
Rheumatic and valvular heart disease (rheumatic)	50 (18.1)	50 (16.4)	57 (13.2)	0.18	716 (14.8)	719 (14.8)	802 (12.1)	<0.001
Interventions, n (%)								
History/index PCI	9 (3.3)	31 (10.2)	33(7.6)	0.03	287 (5.9)	356 (7.3)	530 (8.0)	<0.001
History/index CABG	10 (3.6)	16 (5.2)	20 (4.6)	0.64	291 (6.0)	285 (5.8)	298 (4.5)	<0.001
History/index coronary angiography	96 (34.8)	144 (47.2)	186 (43.1)	0.01	1,909 (39.2)	2,166 (44.5)	3,213 (48.4)	<0.001
Charlson index, n (%)								
0	3 (1.1)	3 (1.0)	0		34 (0.7)	28 (0.6)	37 (0.6)	
1–2	121 (43.8)	98 (32.1)	128 (29.6)	0.001	2,269 (46.7)	2,347 (48.2)	2,975 (44.8)	0.01
3–4	54 (19.6)	82 (26.9)	126 (29.2)		1,323 (27.3)	1,248 (25.6)	1,805 (27.2)	
>4	98 (35.5)	122 (40.0)	178 (41.2)		1,229 (25.3)	1,249 (25.6)	1,822 (27.4)	
Emergency admissions, n (%)	265 (96.0)	293 (96.1)	414 (95.8)	0.99	4,544 (93.6)	4,569 (93.8)	6,275 (94.5)	0.08
Crude cumulative mortality								
30-day case fatality^c^	13 (4.7)	16 (5.3)	19 (4.6)	0.91	332 (6.8)	350 (7.2)	414 (6.4)	0.20
1-year mortality^d^	38 (13.8)	49 (16.1)	55 (17.5)	0.46	1,059 (21.8)	1,018 (20.9)	974 (19.7)	0.04

Continuous variables expressed as mean ± standard deviation (SD). Categorical variables expressed as count (n) and proportion (%)

^a^Patients could have multiple comorbidities. *COPD* = chronic obstructive pulmonary disease

^b^All ischaemic heart disease includes acute myocardial infarction

History/index *PCI* percutaneous coronary interventions as history or concurrent with index HF admission

History/index *CABG* coronary artery bypass graft as history or concurrent with index HF admission

^c^Cases stopped at 30 November 2009 to all 30-day follow-up in December 2009

^d^Cases stopped at 31 Dec 2008 to allow 1-year follow-up in 2009 for the 2008 cases

### Socio-economic status and geographical classification

Socio-economic Indices for Areas (SEIFA) [26] based on residential postcodes and divided into quintiles (based on pre-defined cut-points) were assigned to each patient, as a proxy for socio-economic status. SEIFA was developed by the Australian Bureau of Statistics and ranks areas in Australia according to relative socio-economic advantage and disadvantage. The indexes are based on information from the five-yearly Census, which indicate the collective socio-economic characteristics living in a particular area. The Index of Relative Socio-Economic Disadvantage was used in our analysis [26].

The Accessibility/Remoteness Index of Australia (ARIA) [27] classification and the greater Perth metropolitan city definition [28] were used to dichotomise place of residence into urban and rural.

### Ethics approvals

Ethics approvals were obtained from the WA Aboriginal Health Ethics Committee and the Human Research Ethics Committees of the Western Australian Department of Health and the University of Western Australia.

### Statistical analysis

Three calendar periods of 2000–2002 (baseline period), 2003–2005, and 2006–2009 were used to describe and test for trends. Trends in demographics, comorbidities, interventions, and prevalence of risk factors were assessed separately and compared between Aboriginal and non-Aboriginal HF patients using ANOVA for age and Cochrane-Armatage trend tests for categorical variables.

For 30-day survival, we included all incident patients admitted between January 2000 and November 2009, with December 2009 used as the follow-up period for the November 2009 cases. Survival to 1 year was restricted to 30-day survivors from the 2000–2008 cohort. Risk-adjusted multivariable logistic and Cox regression models were fitted to test for trends in 30-day and 1-year mortality (in 30-day survivors) across the periods and the Aboriginal status and period interaction was used to compare trends for Aboriginal and non-Aboriginal patients. Risk factor adjustment included: age, gender, period, Charlson comorbidity index, specific comorbidities including hypertension, atrial fibrillation, rheumatic/valvular heart disease, diabetes, chronic kidney disease/renal failure, AMI, other IHD, cerebrovascular disease, and history of PCI/CABG. Additionally, PCI and CABG during index admission were modelled for 1-year mortality in 30-day survivors only. Statistical analyses were undertaken with SAS 9.3 and STATA 12.0 for Windows.

## Results

Of 17,379 patients with first HF hospitalization, 1,013 (5.8 %) were Aboriginal. Compared to non-Aboriginal HF, Aboriginal patients had a higher proportion of women and were younger (mean age 54 years vs 71 years non-Aboriginal) (Table 1) with mean age remaining 16–18 years lower at first HF hospitalization over the period. HF presentations by rural Aboriginal patients declined whilst urban presentations increased, but both were not-significant. There was no change in non-Aboriginal patients. Although about half the Aboriginal patients were from areas in the lowest quintile of social disadvantage, the proportion of Aboriginal patients in the lowest (1st) quintile fell significantly (8.6 %) overall (*p* = 0.04). The proportion with private medical insurance increased significantly in non-Aboriginal patients but no change was evident in Aboriginal patients where less than 2 % had private health insurance. A declining trend in length of stay during the index HF hospitalisation was seen in both populations, although not statistically significant in the Aboriginal group.

### Trends in risk factors and HF antecedents

**Fig. 1:**
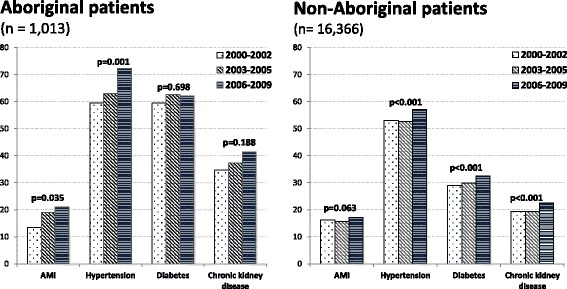
Trends in prevalence of specific risk factors for heart failure in Aboriginal and non-Aboriginal patients, Western Australia 2000–2009

The prevalence of IHD showed a small but significant decline in non-Aboriginal patients [51.8 % (2000–2003), 45.3 % (2006–2009) *p* < 0.001] whereas IHD remained stable in Aboriginal patients (*p* = 0.88) (Table 1). The prevalence of AMI and hypertension, two key antecedents of HF, increased more markedly in Aboriginal than non-Aboriginal patients over the 10-years (Fig. 1). Specifically, the upward trend for AMI was greater in Aboriginal (from 13.4 % to 21.1 %, *p* = 0.04) than non-Aboriginal patients (from 16.3 % to 17.2 %, *p* = 0.06). In all time periods, the prevalence of diabetes and chronic kidney disease was double in Aboriginal patients and increased in both populations over the study period, although not significantly so in Aboriginal patients. Similarly, the prevalence of renal failure was 4-fold higher in Aboriginal patients throughout. Trends in unstable angina, cerebrovascular disease and chronic obstructive pulmonary disease showed significant declines in non-Aboriginal patients only (Table 1). Prevalence of rheumatic/valvular heart disease showed a decline in both subpopulations (though not significantly in Aboriginal patients), suggesting some success in rheumatic heart disease prevention.

In terms of composite comorbidity burden, the change in distribution of the Charlson index over the periods was significantly different in Aboriginal and non-Aboriginal patients (three-way interaction between Charlson index, Aboriginality and period *p* = 0.01). The proportion with a Charlson index score ≥3 increased over time in Aboriginal patients, but was relatively stable in non-Aboriginal patients, with no clinically important trend. Uptake of PCI and coronary angiography increased significantly in both populations, peaking during 2003–2005 in Aboriginal patients. Acute emergency presentations for HF occurred in the vast majority (>94 %), with no significant change over the period.

### Trends in mortality

Crude 30-day mortality did not change over the study period in either population (Table 1). Risk-adjusted 30-day mortality was also not significantly different between the base and last time periods in both Aboriginal (OR 0.98, 95 % CI 0.46-2.08, *p* = 0.98) and non-Aboriginal patients (OR 0.93, 95 % CI 0.80-1.09, *p* = 0.38).

Crude 1-year mortality showed a non-significant increase over the period in Aboriginal patients (p-trend = 0.46), but decreased slightly from 21.8 % to 19.7 % in non-Aboriginal patients (p-trend = 0.04). Risk-adjusted 1-year mortality (Table 2) was non-significantly higher (HR 1.44 95 % CI 0.85-2.41) in Aboriginal patients in 2006–2008 compared with 2000–2002 (p-trend = 0.47), whereas it declined significantly (HR 0.87 95 % CI 0.78-0.97) in non-Aboriginal patients (p-trend =0.01). Independent predictors associated with increased hazards of 1-year adjusted mortality have been reported previously [18] and listed in Table 2. Increasing age, rural residence, lack of private medical insurance, emergency admission, history of CKD/renal failure, AMI and COPD were other independent predictors in non-Aboriginal patients and the whole cohort (data not shown). Notably, the weighted Charlson comorbidity index was the strongest independent predictor of increased hazard (by 26 % for an increase in one unit) in Aboriginal patients.

**Table 2:** Stratified multivariable Cox regression models for 1-year mortality in 30-day survivors, Aboriginal and non-Aboriginal patients

	Non-Aboriginal patients			Aboriginal patients		
	Hazard ratio (HR)	95 % CI	*p*-value	Hazard ratio (HR)	95 % CI	*p*-value
Age	1.04	1.03–1.05	<0.001	1.00	0.99–1.02	0.62
Female sex	0.89	0.81–0.97	0.010	0.97	0.64–1.45	0.87
2000–2002	1.00			1.00		
2003–2005	0.94	0.85–1.05	0.272	1.21	0.71–2.07	0.479
2006–2008	0.87	0.78–0.97	0.010	1.44	0.85–2.41	0.149
Metropolitan residence	1.00			1.00		
Rural residence	1.16	1.03–1.31	0.012	0.75	0.41–1.38	0.354
No private medical insurance	1.15	1.04–1.27	0.007	0.88	0.12–6.62	0.904
SEIFA - 1st quintile (least disadvantaged)	1.00			1.00		
2nd quintile	0.97	0.76–1.24	0.793	0.69	0.31–1.54	0.363
3rd quintile	0.98	0.77–1.24	0.844	0.37	0.18–0.75	0.006
4th quintile	1.03	0.80–1.32	0.837	0.70	0.32–1.53	0.372
5th quintile	1.07	0.83–1.38	0.606	0.84	0.24–2.95	0.787
Emergency admission	1.54	1.18–2.00	0.001	1.61	0.38–6.75	0.515
Comorbidities						
Hypertension	0.83	0.75–0.91	<0.001	0.41	0.25–0.68	<0.001
Atrial fibrillation	0.96	0.87–1.04	0.313	0.96	0.57–1.62	0.885
Rheumatic heart disease	1.28	1.00–1.65	0.051	0.31	0.07–1.35	0.119
Diabetes^a^	0.72	0.64–0.80	<0.001	1.00	0.56–1.80	0.999
Chronic kidney disease^a^	1.24	1.11–1.39	<0.001	0.64	0.37–1.11	0.116
Renal failure^a^	1.36	1.10–1.69	0.005	1.54	0.85–2.80	0.157
Acute myocardial infarction	1.32	1.17–1.48	<0.001	1.55	0.91–2.64	0.109
Ischaemic heart disease	0.87	0.79–0.96	0.006	1.08	0.68–1.71	0.754
Cerebrovascular disease	1.01	0.89–1.15	0.848	1.21	0.63–2.35	0.567
Charlson comorbidity index	1.20	1.18–1.23	<0.001	1.26	1.14–1.39	<0.001
All valvular heart disease	1.02	0.92–1.12	0.745	1.10	0.65–1.87	0.719
COPD	1.11	1.00–1.22	0.047	1.01	0.66–1.54	0.962
PCI (history or 1st admission)	0.66	0.54–0.80	0.000	0.17	0.04–0.73	0.017
CABG (history or 1st admission)	0.65	0.51–0.83	0.001	0.37	0.09–1.60	0.186

^a^Also included in computation of the Charlson comorbidity index

## Discussion

Assessment of modifiable risk factors and outcome trends are important for evaluating key drivers and patterns of disease in the populations and for informing health policy. Over the decade (2000–2009), we observed that key conditions associated with the development of HF increased in the Aboriginal HF patients. Recurrent AMI, hypertension and diabetes are some of the most important risk factors for developing HF [29, 30]. Over the study period, the marked increase in trends of AMI and hypertension in Aboriginal patients and the disproportionate prevalence of diabetes and chronic kidney disease highlight their significantly elevated burden of vascular risk. Of note is the declining trend in RHD among Aboriginal HF patients. Apart from these specific diseases, Aboriginal people with HF also have a substantial burden of other serious conditions (Charlson score ≥3) which increased over the study period. With respect to outcomes, our study indicates that despite advances in medical therapies, the 1-year adjusted survival (in 30-day survivors) following first HF hospitalization did not improve in Aboriginal patients over the 10-year period, contrasting with the significant improvement seen in non-Aboriginal patients and previously reported for the Western Australian population [13].

Aboriginal patients are however presenting at an older age for first HF hospitalization, with less presentations in those aged 20–34 years, suggesting some success in primary prevention programs. There was also a significant reduction the proportion of Aboriginal patient in the most disadvantaged quintile of area-level indices. While IHD is still a key contributor to HF, coronary angiography uptake has increased and was similar in both populations. These results indicate that any socio-economic and health service access gains made by the Aboriginal population (as reflected by access to angiography and RHD prevalence) is not yet reflected in this cohort and that evidence of progress towards reducing HF antecedents and outcomes may only be observed in the future.

In aggregate terms, trends in CVD risk factors for HF point to an increased risk of HF for the Aboriginal group, despite being considerably younger. The substantial social inequities between Aboriginal compared with non-Aboriginal patients (with SEIFA and private medical insurance status as proxies) play a big role in the burden of CVD generally, despite the small but significant decline in the lowest quintile of socio-economic disadvantage in Aboriginal patients over the study period. The reasons for the adverse trends in precursors to HF may also include suboptimal secondary prevention of HF antecedents and poorer post-discharge care and follow-up.

The widening of the gap in one-year outcomes reported in the current study reflects challenges in secondary prevention of HF in Aboriginal patients. Implementation and delivery of guideline-based HF care for rural Aboriginal patients (where the majority reside) is complex [31], challenged by socio-economic, language and cultural factors, poor access to specialist services, poorer access to primary health care [32] and lower participation in post-discharge programs [31]. Despite the higher prevalence of HF in rural areas, lower rates of prescription of evidence-based therapy (e.g. ACE inhibitor drugs), echocardiography for diagnosis and specialist referral have been reported in rural (vs metropolitan areas) [33]. Indeed, a recent study in 3 Northern Territory communities found that 65 % of patients identified with heart failure were previously undiagnosed [34]. Furthermore, difficulties with recruitment and retention of adequately trained health professionals in rural/remote areas render current integration and continuity of care for Aboriginal patients (many with multimorbidity), woefully inadequate [35, 36].

HF usually reflects the end stage of underlying heart conditions, so that HF prevention includes primary prevention of these conditions. Our results suggest gains in primary prevention of CVD in Aboriginal people are lagging, with further management of key antecedents of HF being inadequate. Data from our previous study [18] showed widening (adjusted) disparities after the first month post-discharge for Aboriginal patients. Compounding this picture of health inequalities, Aboriginal patients who survive a major coronary event are also less likely (than non-Aboriginal patients) to attend a cardiac rehabilitation programs with location of such services in predominantly urban and major regional centres [37], reflecting inequalities in health services access in the community. Social, policy and systems change are needed across multiple sectors to reduce the Aboriginal social disadvantage contributing to adverse risk factor trends (of antecedents) and barriers to accessing health services. There is an urgent need for environments that facilitate healthy choices to reduce risk factors contributing to premature HF. Furthermore, primary health care (as the cornerstone of health services system) need to be strengthened and access to cardiac rehabilitation improved for Aboriginal people in WA.

The risk factor trends suggest potential differences in epidemiological transition of HF between both populations, at least for rheumatic heart disease, for which Aboriginal people still have very high rates [38]. The declining trend in RHD may be due to progress from coordinated programs targeted at improved detection, monitoring, management and prophylaxis of acute rheumatic fever/rheumatic heart disease in the Aboriginal community [38].

Although this study is specific to Aboriginal Australians in Western Australia, Aboriginal Australians share similar clinical, socio-demographic and historical characteristics with other Indigenous populations around the world [3]. Furthermore, epidemiological findings from one region or a particular ethnic group are likely to be relevant to another region or ethnic group [39].

Unfavourable trends in some key risk factors for cardiovascular diseases were similarly reported to underlie the slowing of the decline in IHD deaths among Aboriginal Australians in the younger age groups [40]. Separately, we also observed a two-fold adjusted hazard of 1-year mortality in 30-day survivors in younger Aboriginal Australians (less than 55 years) following incident HF hospitalization [18]. These are consistent with the findings from a Canadian study, which reported that the relative risk of dying from CVD for First Nations people (compared to non-Aboriginal cohort) was highest in the younger age groups (25–34 years) [41]. Other studies from Canada showed a 2.5-fold higher prevalence of CVD in Aboriginal people (vs non-Aboriginal people of European descent) [3], similar IHD mortality rates among Aboriginal and Canadian males, but 61 % higher IHD mortality among Aboriginal women compared with Canadian women [39]. A more recent study from Alberta also observed 18 % to 39 % higher adjusted mortality at 1-year and 5 years, respectively, in Aboriginal (vs non-Aboriginal) patients following index HF hospitalization [8].

The strengths of this study lie in the quality and near complete ascertainment of the short-term and 1-year mortality following the index hospitalisation for HF using the WA HMDC [19] and the validation of a principal diagnosis of HF [20]. However, we do not have medication records, echocardiography findings and clinical data recorded within administrative data. Furthermore, there are unrecorded factors (such as smoking, diet and alcohol use) affecting Aboriginal health and environmental factors which could adversely affect health outcomes. The use of an area-based measure (SEIFA), as an indicator for social deprivation, could give rise to potential misclassification at an individual level.

## Conclusions

This population-based cohort study showed widening disparities between Aboriginal and non-Aboriginal patients with HF in both key individual risk factors and composite comorbidity index. The mortality trends highlight that gains in secondary prevention in the mainstream population have not been achieved in the younger, Aboriginal population. This disparity warrants urgent policy attention particularly around prioritising better prevention of heart disease, enhanced surveillance for heart disease and management of antecedents in primary care. Since up to 80 % of premature cardiovascular disease is preventable, it is possible to substantially reduce this disproportionate burden of CVD in Aboriginal Australians through addressing the underlying material, social and environmental factors associated with disadvantage. These disadvantages are a significant barrier to the effectiveness of medical interventions [42], so upstream interventions to reduce risk factors will also support efforts directed at improving treatment of HF. Since primary care plays a central role in mitigating CVD in the population, strenuous efforts to strengthen primary health care and improve access to cardiac rehabilitation, particularly outside of metropolitan areas, are needed to complement population prevention measures.

## Abbreviations

CVD: Cardiovascular disease
HF: Heart failure
IHD: Ischaemic heart disease
WA: Western Australia
HMDC: Hospital morbidity data collection
AMI: Acute myocardial infarction
PCI: Percutaneous coronary intervention
CABG: Coronary artery bypass grafting
SEIFA: Socio-economic indices for areas
